# Phlorotannins of the Brown Algae *Sargassum vulgare* from the Mediterranean Sea Coast

**DOI:** 10.3390/antiox11061055

**Published:** 2022-05-26

**Authors:** Amina Chouh, Tahar Nouadri, Marcelo D. Catarino, Artur M. S. Silva, Susana M. Cardoso

**Affiliations:** 1 Laboratory of Microbiological Engineering and Application, Department of Biochemistry and Molecular and Cellular Biology, Faculty of Nature and Life Sciences, University of Mentouri Brothers Constantine 1, Constantine 25017, Algeria; chouh.amina@umc.edu.dz (A.C.); nouadri.tahar@umc.edu.dz (T.N.); 2 Biotechnology Research Center CRBT, Constantine 25016, Algeria; 3 LAQV-REQUIMTE & Department of Chemistry, University of Aveiro, 3810-193 Aveiro, Portugal; mcatarino@ua.pt (M.D.C.); artur.silva@ua.pt (A.M.S.S.)

**Keywords:** *Sargassum*, antioxidant, antidiabetic, pancreatic lipase, anti-inflammatory, UHPLC-MS analysis

## Abstract

Brown seaweeds are a good source of bioactive compounds, particularly of phlorotannins, which may exert a wide spectrum of pharmacological properties. In the present study, phlorotannins of *S. vulgare* were extracted using a 70% acetone solution and the crude extract was further purified through liquid–liquid partition, giving rise to *n*-hexane, ethyl acetate and aqueous residue fractions. The crude extract and the purified fractions were evaluated for potential antioxidant abilities as well as for inhibitory potential towards the digestive enzymes α-amylase and pancreatic lipase, and anti-inflammatory potential through the hindering of albumin denaturation. Overall, the ethyl acetate fraction was the richest in phlorotannins (9.4 ± 0.03 mg PGE/g) and was also the most promising regarding the tested bioactive properties. Of note, its inhibitory potential towards α-amylase was about nine times that of the commercial drug acarbose and its inhibitory activity against high temperature-induced protein denaturation was superior to that of the non-steroidal drug ketoprofen. According to UHPLC-DAD-ESI-MS/MS analysis, this fraction contained a range of phlorotannins with at least six units of phloroglucinol, including dibenzodioxine-1,3,6,8-tetraol, fuhalol, pentaphlorethol, fucopentaphlorethol and dihydroxypentafuhalol, in addition to several less common phlorotannin sulfate derivatives.

## 1. Introduction

In recent years, marine macroalgae have attracted a great deal of attention as natural sources of multiple biochemically active molecules with valuable applications in numerous industrial fields, including textile, material, cosmetic, biomedical, pharmaceutical and—above all—food industries [1]. Nutritionally, seaweeds represent an important source of minerals (7–36%), such as calcium, iron, copper and iodine; polysaccharides (15–76%), including agar-agar, alginate and carrageenan; proteins (5–47%), containing all the essential amino acids; and lipids (1–5%) particularly high in polyunsaturated fatty acids. Moreover, seaweeds generally have high contents of several micronutrients, such as vitamins A, B1, B12, C, D and E, as well as a huge variety of phenolic compounds, such as phlorotannins, bromophenols, flavonoids, phenolic terpenoids and mycosporine-like amino acids [2,3,4].

Among the different types of seaweed, members of the genus *Sargassum* (class: Phaeophyceae, order: Fucales, family: Sargassaceae) have been associated with multiple health benefits. For example, *S. fusiforme*, one of the most renowned species of this genus, along with *S. thunbergii*, *S. wightii*, *S. muticum* and others, have been claimed to exert anticancer, antiangiogenic, antimicrobial, antioxidant and anti-inflammatory effects, among others [5,6,7]. In this field, *S. vulgare* is no exception. Different extracts of *S. vulgare* origin (ethanol, methanol, diethyl ether, aqueous and others) have been described as antimicrobial against several bacteria, including multi-drug resistant *Staphylococcus aureus* and *Aeromonas hydrophila* [8,9,10], antiviral against human immunodeficiency and hepatitis C virus [11] and antiparasitic against *Trypanosoma brucei* [10]. Moreover, strong antitumor properties have been frequently demonstrated for polysaccharides isolated from *S. vulgare*. Indeed, Dore et al. [12] found that the incubation of rabbit aorta endothelial cells with sulphated polysaccharides of *S. vulgare* origin significantly inhibited the secretion of vascular endothelium growth factor, thus inhibiting tubulogenesis and angiogenesis. The authors also found that a significant antiproliferative action (47%) on HeLa tumor cells occurred upon incubation with these polysaccharides. In a different study, the oral administration of alginate isolated from this species to sarcoma 180 cell-transplanted mice was found to inhibit tumor proliferation by more than 50% [13]. Other bioactive properties described for *S. vulgare* polysaccharides include anticoagulant and antithrombotic, antioxidant, immunostimulatory, anti-inflammatory and hypolipidemic activities [14,15,16].

In turn, the health benefits of phenolic-rich extracts, more specifically, phlorotannins, i.e., brown algae-exclusive polyphenols formed through the C–C and/or C–O–C oxidative coupling of phloroglucinol (1,3,5-trihydroxybenzene), is a topic that remains quite underexplored [17]. In this field, the most thoroughly addressed subject is the antioxidant activities of phenolic extracts, which has been studied using different solvents (methanol, acetone, dichloromethane:methanol) and radical scavenging assays that have been screened for specimens collected in the Atlantic Ocean Coast of Brazil and in the Adriatic Coast of Montenegro [11,18,19,20]. More recently, Arunkumar et al. [21] reported an interesting anti-hyaluronidase activity for a phlorotannin extract obtained from *S. vulgare* from the Arabic Sea off the coast of India. Notably, no phlorotannin characterization has been provided so far for this species.

Interestingly, although *S. vulgare* is widely disseminated in the tropical and subtropical regions of the Atlantic, with great prominence around the Azores, Madeira and Canary Islands and in the Mediterranean Sea [22], little is known about the diversity of *S. vulgare* in Algeria, which has a littoral stretching over 1200 km. To our knowledge, the few existing studies that have focused on Algerian *S. vulgare* have only been carried out in some areas of Algeria’s central coasts (Bordj El Kiffan, Tamentfoust, Surcouf, Sidi Fredj, Arzew, Cherchell, Ain Tagourait, Kouali, Bou-Ismail, Ain Benian, Bourmedes, Gouraya, Taza) [23,24,25].

In this context, this study aims to evaluate the chemical composition of *S. vulgare* and examine its phlorotannin content in detail, as well as to evaluate the bioactive potential of crude and purified phlorotannin-rich extracts from *S. vulgare* grown in northeast Algeria (Gulf of Stora, Mediterranean Sea), focusing on the antioxidant, anti-inflammatory, antidiabetic and anti-obesity activities.

## 2. Materials and Methods

### 2.1. Solvents and Reagents

Butylated hydroxyanisole (BHA), butylated hydroxytoluene (BHT), 1,1-diphenyl-2-picrylhydrazyl (DPPH), α-tocopherol, ascorbic acid, β-carotene, linoleic acid, polyoxyethylene sorbitan monopalmitate (Tween-40), neocuproine, galvinoxyl, potassium ferricyanide, trichloroacetic acid, starch powder, iodine, 2,2′-azino-bis(3-ethylbenzothiazoline-6-sulfonic acid), diammonium salt (ABTS), Acarbose (≥95%), potassium iodide, α-amylase from *Aspergillus oryzae* Green Alternative powder, ≥150 units/mg protein (biuret), *p*-nitrophenyl palmitate (*p*NPP), lipase from porcine pancreas, Bovine serum albumin (BSA) and orlistat were obtained from Sigma Chemical Co. (Sigma-Aldrich GmbH, Sternheim, Germany). Ketoprofen^®^, sodium carbonate, iron(III) chloride (FeCl_3_), sodium bicarbonate, copper(II) chloride, phenanthroline, potassium persulfate and ammonium acetate were obtained from Biochem Chemopharma. Formic acid and 2,4-dimethoxybenzaldehyde (DMBA) were purchased from Sigma (St. Louis, MO, USA). Acetonitrile, hydrochloric acid and glacial acetic acid were acquired from Fisher (Pittsburgh, PA, USA). Solvents, including ethanol and methanol of high-performance liquid chromatography (HPLC) purity, were purchased from Lab-Scan (Lisbon, Portugal). 

### 2.2. Seaweed Samples

**Figure 1:**
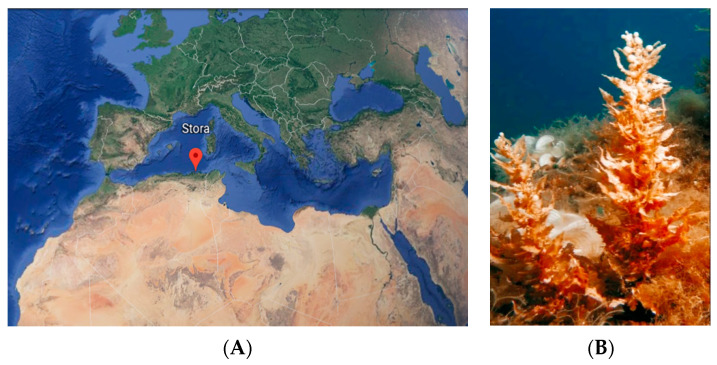
Overall illustration of sampling site at Stora, Skikda, Algeria, 36°53′54.9″ N, 6°52′48.1″ E (**A**) and *S. vulgare* (**B**).

*S. vulgare* (≅1 kg) was collected in February 2018, at Stora, Skikda, in northeastern Algeria (Figure 1), latitude: 36°53′54.9″ N, Longitude: 6°52′48.1″ E. The parameters of water quality detected via a multiparameter meter (HI98194, Hanna, Romania) were salinity (35.08 PSU), conductivity (15.22 ms/cm) and pH (8.16). The samples were immediately washed with seawater to remove possible debris, adhering sand particles and epiphytes, and then transported to the laboratory in bags and washed with tap water to eliminate any presence of salt. A part of the cleaned seaweeds was milled with a microfine grinder (IKA, MF 10, Germany) and chemical characterization was performed as described in Section 2.3. The remaining macroalgae was lyophilized, milled and stored in the freezer (−20 °C) until used for analysis, as described in Section 2.4. 

### 2.3. Chemical Characterization of S. vulgare

The major biochemical components, i.e., protein, carbohydrate, fat, fibre and ash contents, were determined according to the Association of Official Analytical Chemists methods [26]. Total carbohydrate content was determined by difference using the equation:

Carbohydrates = 100 − (% Ash) − (% Total Fat) − (% Protein)

### 2.4. Obtainment of Phlorotannin Extract and Purification

Phlorotannins were obtained with hydroacetone mixture, as previously described by Catarino et al. [27]. For this, the algae powder (30 g) was dispersed in 2.1 L of 70% acetone solution (*v*/*v*) at room temperature for 3 h under constant agitation. The mixture was filtered through a G4 sintered plate filter, and the resulting solution was concentrated in a rotary evaporator to a final volume of approximately of 250 mL. The concentrated extract solution was sequentially partitioned several times with *n*-hexane (1:1, *v*/*v*), until a colorless nonpolar fraction was obtained, and the aqueous phase was further submitted to liquid–liquid extraction, three times, with ethyl acetate (1:1, *v*/*v*). The resulting fractions (*n*-hexane, ethyl acetate and aqueous residue) were stored at −20 °C until analysis.

### 2.5. Characterization of Phlorotannins 

Quantification of total phlorotannins was carried out for the crude extract and the purified fractions according to the 2,4-dimethoxybenzaldehyde (DMBA) colorimetric method, as previously described [28]. The working reagent was prepared by mixing equal volumes of DMBA (2%, *m*/*v*) and hydrochloric acid (6%, *v*/*v*), both prepared in glacial acetic acid. Then, 50 μL of each extract was mixed with 250 μL of working reagent at room temperature for 1 h. The absorbance was determined at 515 nm using a microplate reader), EnSpire, PerkinElmer, Waltham, MA, (US) United States of America. The phlorotannin content was determined by using a regression equation of the phloroglucinol linear calibration curve (0.06–0.1 mg/mL).

More detailed information about the phlorotannins’ constituents was obtained from the acetate ethyl fraction, using UHPLC-ESI-DAD-MS^n^ analysis, as previously described by Catarino et al. [27]. This analysis was carried out in an Ultimate 3000 (Dionex Co., San Jose, CA, USA) apparatus consisting of an autosampler/injector, a binary pump, a column compartment and an Ultimate 3000 Diode Array Detector (Dionex Co., San Jose, CA, USA) coupled to a Thermo LTQ XL (Thermo Fisher Scientific, San Jose, CA, USA) ion trap mass spectrometer equipped with an ESI source.

The mobile phase was composed of (A) 0.1% (*v*/*v*) acetonitrile and (B) formic acid, following the general procedure previously described in [29]. The solvent gradient started with 5–40% of solvent (A) over 14.72 min, from 40–100% over 1.91 min and remaining at 100% for 2.19 min before returning to the initial conditions. The flow rate was 0.2 mL min^−1^ and UV–Vis spectral data for all peaks were accumulated in the range of 200–600 nm, while the chromatographic profiles were recorded at 280 nm. Control and data acquisition were carried out with the Thermo X calibur Qual Browser data system (Thermo Fisher Scientific, San Jose, CA, USA). Nitrogen above 99% purity was used, and the gas pressure was 520 kPa (75 psi). The instrument was operated in the negative-ion mode with ESI needle voltage set at 5.00 kV and an ESI capillary temperature of 275 °C. The full scan covered the mass range from *m*/*z* 100 to 2000. CID-MS/MS and MS^n^ experiments were simultaneously acquired for precursor ions using helium as the collision gas with a collision energy of 25–35 arbitrary units.

### 2.6. Antioxidant Activities

The antioxidant capacities of the crude extract and of the purified fractions were estimated through distinct in vitro tests, namely, 2,2-diphenyl-1-picrylhydrazyl (DPPH^•^), ABTS^•+^, superoxide, galvinoxyl, cupric reducing antioxidant capacity (CUPRAC), reducing power (FRAP), phenanthroline and β-carotene bleaching assays. In all cases, the potency of the sample was compared with that of standard antioxidant compounds (BHA, BHT, α-tocopherol and ascorbic acid).

#### 2.6.1. DPPH^•^ Scavenging Test 

Scavenging activity against DPPH free radicals was assessed according to the method described in [30]. Briefly, methanolic DPPH solution (0.1 mM) was mixed to various concentrations of samples and incubated for 30 min at room temperature in the dark. The absorbance was measured at 517 nm using a microplate spectrophotometer (PerkinElmer, EnSpire, Waltham, MA, USA) and the results were expressed as IC_50_ values, i.e., the concentration of sample capable of 50% inhibition. 

#### 2.6.2. ABTS^•+^ Scavenging Activity

ABTS^•+^ scavenging activity was determined following the procedure described by Nickavar et al. [31]. A reaction between 7 mM ABTS and 2.45 mM potassium persulfate generated the cation ABTS^•+^. After 24 h, the absorbance of ABTS solution was adjusted to achieve an absorbance of 0.700 ± 0.020 at 734 nm. After that, 160 μL of ABTS^•+^ solution was mixed with 40 μL of extracts at different concentrations. The absorbance was measured at 734 nm after 10 min in the dark. The results were expressed as IC_50_ values.

#### 2.6.3. Cupric Reducing Antioxidant Capacity (CUPRAC) 

This test followed a previously reported method [32]. In a 96-well plate, a volume of 50 μL of each sample at different concentrations (3.1–200 μg/mL) was mixed with 50 μL of CuCl_2_ (10 mM), 50 μL of neocuproine (7.5 mM) and 60 μL of ammonium acetate buffer (NH_4_Ac, 1 mol/L, pH 7.0). The absorbance was then measured at 450 nm after 1 h of incubation at room temperature. The results were expressed as A_0.50_ values (i.e., concentration required to produce 0.5 absorbance). 

#### 2.6.4. β-Carotene Bleaching Assay

Lipid peroxidation inhibition activity was evaluated using the β-carotene–linoleic acid bleaching assay [19]. An emulsion solution of β-carotene was mixed with 40 μL of the sample at different concentrations (12.5–800 µg/mL). The absorbance was measured at 470 nm, 0 min and 120 min after incubation for 120 min at 50 °C. The results were given as IC_50_s, obtained by the following formula:

Inhibition (%) = [(AE(t = 0) − AE(t = 120))/(AC(t = 0) − AC(t = 120))] × 100

where AE(t = 0) and AE(t = 120) are the absorbances of the tested samples at 0 min and 120 min, respectively, while AC(t = 0) and AC(t = 120) are the absorbances of the negative control measured at 0 min and 120 min, respectively.

#### 2.6.5. Superoxide Radical Scavenging Activity

Superoxide radicals (O_2_^•−^) were generated according to the alkaline DMSO method using nitroblue tetrazolium (NBT) [33]. The reduction of NBT to formazan by superoxides can be measured at 560 nm. The results were expressed as IC_50_s.

#### 2.6.6. Galvinoxyl Radical (GOR) Scavenging Assay

Galvinoxyl radical scavenging was determined according to the method described in [32]. Fresh galvinoxyl radical solution (0.1 mM) was prepared in methanol and mixed with 40 μL of sample. The absorbance was measured at 428 nm after incubation at room temperature for 120 min. The results were expressed as IC_50_s.

#### 2.6.7. Reducing Power Assay

The reducing power assay was performed with potassium ferricyanide according to Catarino et al. [30] and the absorbance was measured at 700 nm. Different concentrations of extracts were mixed with 0.2 M phosphate buffer (pH 6.6) and 1% of potassium ferricyanide K_3_Fe(CN)_6_ after incubation at 50 °C for 20 min. Then, 10% of trichloroacetic acid and 10 μL ferric chloride FeCl_3_ (0.1%) were added. The results were expressed as A_0.50_s (i.e., concentration producing 0.5 absorbance).

#### 2.6.8. Phenanthroline Assay 

Phenanthroline assays were performed according to [34]. For this assay, 10 µL of extract at different concentrations was added to 50 µL of 0.2% ferric chloride FeCl_3_ solution and 30 µL of 0.5% phenanthroline solution. The reaction mixture was made up to 200 µL with methanol and absorbance was measured at 510 nm after incubation at 20 min/30 °C. The results were given as absorbance levels (A_0.5_ µg/mL). 

### 2.7. Enzymatic Assays

#### 2.7.1. α-Amylase Inhibition Assay

The α-amylase inhibitory activity of *S. vulgare* was evaluated using the iodine/potassium iodide (IKI) method, according to [35]. Briefly, 25 µL of different extract concentrations (or acarbose) was mixed with α-amylase solution (50 µL) dissolved in phosphate buffer (pH 6.9, containing 6 mM of NaCl) and then incubated for 10 min at 37 °C. After this, 50 µL of a 0.1% starch solution was added and incubated for 10 min. Finally, 25 µL of HCl (1 M) and 100 µL IKI were added to each well in the 96-well microplate to stop the reaction. The absorbance was read at 630 nm using a multimode microplate reader (PerkinElmer, EnSpire, Waltham, MA, USA) and the results were expressed as IC_50_s.

#### 2.7.2. Pancreatic Lipase Inhibition Assay 

Pancreatic lipase inhibitory activity was estimated by means of the method described by Pereira et al. [36], using chromogenic *p*-nitrophenyl palmitate (*p*NPP) as a substrate which than hydrolyzed by lipase to *p*-nitrophenol (*p*NP), a colored agent that can be monitored at 410 nm. Briefly, 50 µL of several concentrations of different extracts (or orlistat) in DMSO was pre-incubated with 100 µL of pancreatic porcine lipase at 37 °C for 20 min. Then, 50 µL of *p*-nitrophenyl palmitate was added to the plate, which was then incubated for 120 min at 37 °C. The results were expressed as IC_50_s. 

### 2.8. Inhibition of Bovine Serum Albumin Denaturation

For this assay, 500 µL of sample solution (at 62.5, 125, 250, 500 µg/mL) were added to 500 µL of 0.2% *w*/*v* BSA (prepared in Tris-buffered saline, pH 6.6). The control consisted of 500 µL of 0.2% BSA solution with 500 µL water and the standard consisted of 100 µg/µL of ketoprofen in water with 500 µL of BSA solution. All the test tubes were incubated at 37 °C for 15 min, then in a water bath at 72 °C for 5 min. The absorbance of these solutions was determined using a UV–Vis spectrophotometer (Thermo scientific-Helios Epsilon, Loughborough, UK) at 660 nm. The inhibition against denaturation of BSA was calculated using the following equation:

Inhibition of denaturation% = Abs control − Abs sample/Abs control × 100

### 2.9. Statistical Analysis

All data for all phytochemical studies and biological activity tests are presented as the means of analyses in triplicate. Values are the means of three assays ± standard deviation. The one-way ANOVA test (XLSTAT) was used to statistically compare the mean values for each treatment. All the antioxidant and enzymatic tests were carried out at more than four concentration values. The IC_50_ and A_0.50_ values were calculated by linear regression analysis, and one-way analysis of variance ANOVA was used to detect significant differences (*p* < 0.05).

## 3. Results and Discussion 

### 3.1. Chemical Composition of S. vulgare

According to the literature, brown algae are essentially composed of carbohydrates, which make up around 50–60% of the dried weight (DW), while protein, fat, fiber and ash contents are in the ranges of 3–15%, 1–3%, 10–62% and 21.1–39.3%, respectively [37,38,39]. 

In this study, we found that for *S. vulgare* from the Mediterranean Coast carbohydrates represented 68% of its DW (Table 1), a value that is in good agreement with the data previously reported for a specimen collected from Brazil (67.8% DW) [40] and higher than that reported by Arguelles et al. for a *S. vulgare* specimen collected from the Philippines (34.18% DW) [39]. The result of this study also showed a similar carbohydrate content as compared to levels described by Heo et al. in other species, such as *S. fulvellum*, *S. thunbergia* and *S. coreanum* (62.5% DW, 63.6% DW and 67.2% DW, respectively) [41]. However, lower total carbohydrate contents were reported by Vijay et al. in *S. ennerimum* (23.54%) and *S. wightii* (23.50%), respectively [42]. *S. vulgare* also showed a relatively high dietary fiber content (21.1% DW), which is similar to that reported for a sample collected from Batangas Bay in the Philippines [39] and higher than another sample collected from the Red Sea Coast of Jeddah (16.5% DW) [43]. 

The ash content of *S. vulgare* collected from the northeast of Algeria was 23.7% DW, falling between the values reported by Marinho-Soriano et al. [40] and Arguelle et al. [39] (19.4% DW and 27.1% DW, respectively) for *S. vulgare* from Brazil and the Philippines. As expected, low protein and fat contents were observed in this seaweed species, representing 7.7% and 0.51% DW, respectively. Interestingly, the fat content of this species is considerably low compared with the ranges that are usually described in brown seaweeds (1–3%), although the values obtained here are in close agreement with previous data reported in the literature for this species [44,45].

### 3.2. Extraction and Fractionation of Phlorotannins

The acetone 70% system was used to extract phlorotannins since, in general, this is recognized as the most suitable solvent for the extraction of phlorotannins from brown seaweeds [27]. The extraction yield of crude extract of *S. vulgare* represented approximately 27% *w*/*w* (Table 2), a lower value than that obtained by Acevedo Garcia et al. for *S. muticum* collected from Spain (38.3%) [46]. On the other hand, the total phlorotannin content (TPhC) of the crude extract was higher than that described by Lopes et al. [47] (7.50 mg/100 g DW) for *S. vulgare* hydroacetonic extract (1386.5 mg/100 g DW). The same study confirmed that *S. vulgare* contained lower amounts of phlorotannins than the other species in this family. This variability in TPhC is naturally explained not only by environmental factors (e.g., growth conditions, harvesting season, etc.) but also by the methodologies used to treat the samples [48].

The purified fractions generated from the crude extract after solvent partitioning, namely, the ethyl acetate fraction (EtOAc), hexane fraction (Hex) and the aqueous residue (AQ), had a mass yield of 6.1%, 10.8% and 63.0%, respectively. The TPhC in the EtOAc was twice that of the crude extract, thus confirming its additional purity, while the Hex and AQ were poorer in TPhC. The absence of literature data concerning the levels of phlorotannins in purified fractions of *S. vulgare* hampers the making of a direct comparison with our results. Nevertheless, the TPhC values found for the EtOAc are close to those found in *S. fusiforme* (8.8 mg PGE/g extract) [49] and higher than that reported for *S. muticum* collected from the Norwegian sea (5.115 mg PGE/g extract) [50].

### 3.3. Antioxidant Activities 

It is well known that there is a lack of a universal test for assessing antioxidant capacities. Thus, the antioxidant potential of *S. vulgare* extracts/fractions was estimated on the basis of their ability to scavenge the free radicals 2,2-diphenyl-1-picrylhydrazyl (DPPH^•^), ABTS^•+^, superoxide and galvinoxyl, which can occur by a mechanism of electron or hydrogen donation. In addition, cupric reducing antioxidant capacity (CUPRAC), reducing power (FRAP) and phenanthroline assays were used to investigate the samples’ abilities to reduce metallic ions through the electron transfer mechanism (Cu^2+^ to Cu^1+^ and Fe^3+^ to Fe^2+^, respectively), while the β-carotene bleaching assay was applied to evaluate their abilities to inhibit lipid peroxidation. The results are summarized in Table 3.

Overall, EtOAc exhibited considerable antioxidant potential, more promising than the crude extract, Hex and AQ—a fact that is probably associated with its higher phlorotannin content, as previously demonstrated for other seaweeds [51]. In fact, the only exception was observed for the β-carotene assay, in which Hex was shown to be more active. Nevertheless, it should be noted that this method employs an emulsified system, which usually allows non-polar compounds to exhibit a stronger antioxidant effect in emulsions because they concentrate at the lipid phase [31]. Not surprisingly, regardless of Hex had lower levels of phlorotannins as compared to the crude extract, it exerted superior antioxidant activity, which is likely due to the presence of antioxidant pigments, particularly fucoxanthin. The results demonstrate that, like other brown macroalgae species, the hydroacetonic crude extract of *S. vulgare* and/or some its purified fractions possess promising antioxidant properties, some closely associated with the presence of phlorotannins. In fact, this was an expected result, since antioxidant activity is perhaps the most well-documented property of polyphenols in general and phlorotannins are no exception. Nevertheless, up to now, studies on the antioxidant properties of *S. vulgare* have been mainly focused on their sulfated polysaccharides, particularly fucoidans, while phlorotannins remained quite underexplored. However, steps have already been taken to address this shortcoming, with some authors reporting interesting antioxidant properties for phlorotannin extracts of this species. Indeed, different DPPH^•^ scavenging effects were reported for extracts of different organs (stipes vs. blades) of *S. vulgare* [21]. In turn, Martins et al. [19] found that although *S. vulgare* dichloromethane: methanol (1:1) extract presented one of the highest total phenolic compound contents among 26 different macroalgae species tested, it showed one of the lowest inhibitory effects in the β-carotene bleaching assay. It should, however, be noted that the total phenolic content of the extracts was estimated using the Folin–Ciocalteau method, which is not as precise as DMBA for phlorotannins and may lead to overestimations of phenolic concentrations in some samples.

### 3.4. Inhibition of Enzymatic Activity

In addition to the antioxidant potential, we evaluated the ability of *S. vulgare* crude extract and purified fractions to inhibit the activity of α-amylase and pancreatic lipase. These two digestive enzymes are claimed to be effective targets for the treatment of type II diabetes and obesity. In particular, α-amylase catalyzes the hydrolysis of carbohydrates into simple sugars and its inhibition retards the digestion of starch and oligosaccharides, contributing to the reduction of postprandial increases in plasma glucose levels. In turn, lipase inhibition decreases the digestion of dietary triglycerides, hence reducing the levels of free fatty acids and monoacylglycerols in the intestinal lumen [52].

As shown in Table 4, EtOAc and Hex exhibited promising inhibitory capacities against the activity of the two digestive enzymes, being particularly active against α-amylase, as can be observed by comparing their IC_50_ values with that of acarbose (approximately nine times stronger than the pharmaceutical drug). The ability of algae extracts to inhibit the activity of α-amylase has been correlated with the capacity of phlorotannins to act toward this enzyme via protein-biding effects, this most likely being the mechanism described for the interactions between land tannins and proteins in general [53]. The Hex fraction, on the other hand, was not expected to exhibit inhibitory effects as strong as the EtOAc fraction since it is almost devoid of phlorotannins. However, this fraction was found to contain a significant amount of fucoxanthin, which could explain this result, as this carotenoid is also described as a strong inhibitor of α-amylase [54]. 

Although the effects of *S. vulgare* extract/fractions against pancreatic lipase were quite unlike those of orlistat, EtOAc was found to be the most active of the tested samples (IC_50_ of 17.98 ± 2.2 μg/mL). Since saponins, terpenes and polyphenols are well-known to be active against pancreatic lipase [55,56,57,58], it is highly possible that the superior phlorotannin content present in this fraction is the reason for the results observed. Promising inhibitory effects of extracts from other *Sargassum* seaweeds have been previously reported either against α-amylase or pancreatic lipase. Indeed, besides the high potential to inhibit α-amylase, the (hydro)alcoholic extracts of *S. aquifolium* [59], *S. ringgoldianum subsp*, *Coreanum*, *S. siliquastrum*, *S. patens* and *S. piluliferum* were found to exert inhibitory abilities towards α-amylase, with IC_50_s between 30 and 60 μg/mL [60], while inhibitions above 60% were described for methanolic extracts of *S. muticum*, *S. ringgoldianum* and *S. hunbergia* towards pancreatic lipase [61]. Nevertheless, up to now no other studies have focused on the effects of *S. vulgare* extracts towards these two metabolic enzymes.

### 3.5. Anti-Inflammatory Activity

Inflammation is a biological reaction of the body to harmful stimuli, such as bacteria, toxins, pathogens, heat or any other cause. It involves increases in protein denaturation, vascular permeability and membrane alteration, among other effects [62]. In this respect, albumin denaturation is a marker for inflammatory diseases [63]. To evaluate the potential anti-inflammatory activity of *S. vulgare* extract and fractions, we further estimated their ability to inhibit albumin denaturation in vitro.

**Figure 2:**
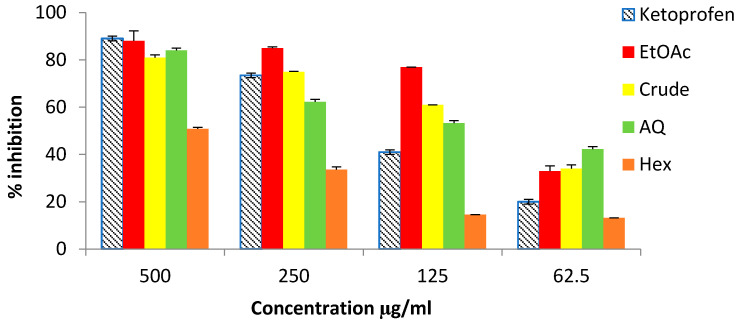
Inhibitory ability of *S. vulgare* crude extract and of purified fractions towards albumin denaturation. Crude—crude extract; Hex—hexane fraction; EtOAc—ethyl acetate fraction; AQ—aqueous residue.

As shown in Figure 2, the crude hydroacetonic extract, as well as the soluble purified fractions, showed a concentration-dependent inhibitory activity against high temperature-induced protein denaturation, with the following order of decreasing effectiveness: EtOAc > Crude > AQ > Hex. Interestingly, the results revealed that for lower concentrations, the effects of the seaweed extract and fractions (with exception of Hex) were even superior to those observed for ketoprofen, which is a non-steroidal drug. At 125 µg/mL, the inhibitions registered for EtOAc (77%), crude extract (61%) and AQ (53%) were approximately two times more effective than the control drug (41%).

The ability of *S. vulgare* extract to reduce the thermal denaturation of proteins is possibly a factor contributing to its anti-inflammatory activity. The therapeutic applications of phlorotannins with respect to inflammation have previously been reported by Lopes et al., who showed that strong direct scavenging activity of sodium nitroprusside (SNP) generated NO^•^ in a cell-free system in *S. vulgare* [47]. However, further experiments would be necessary to understand the real contribution of phlorotannins to the anti-inflammatory results observed. Nevertheless, the data herein presented clearly indicate that *S. vulgare* exerts significant anti-inflammatory activity through inhibition of albumin.

### 3.6. Characterization of Phlorotannins

**Figure 3:**
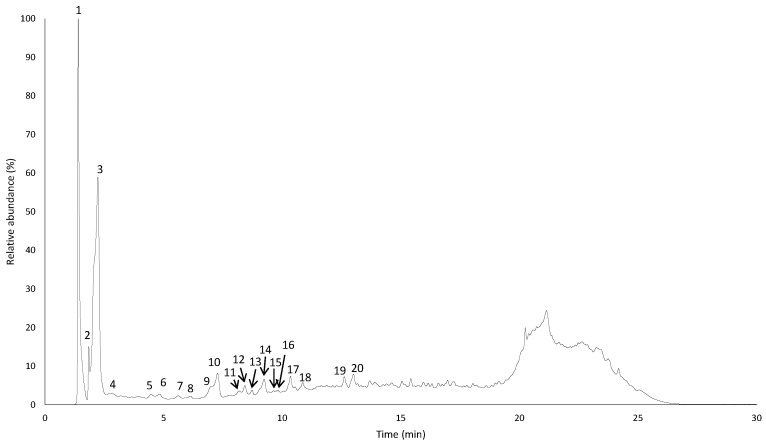
Chromatographic profile of the ethyl acetate fraction of *S. vulgare* extract at 280 nm. Peaks marked with numbers correspond to the tentatively identified compounds represented in Table 5.

As phlorotannins of *S. vulgare* have not yet been revealed, we selected EtOAc (i.e., the richest sample in TPhC) to proceed with the elucidation of its phlorotannin profile. As represented in Figure 3, the UV chromatogram obtained at 280 nm showed a typical shape of a phlorotannin mixture, with an initial region of reasonably well-separated peaks followed by a region of extensive coelution, characterized by an unresolved hump. 

Overall, a total of 21 phlorotannins and derivatives with molecular weights ranging from 2 to 6 units of phloroglucinol were detected in the EtOAc fraction of *S. vulgare* extract (Table 5). Out of these, it was possible to properly characterize five compounds based on their MS^2^ spectra and comparison with data in the literature. These were: dibenzodioxine-1,3,6,8-tetraol ([M − H]^−^ at *m*/*z* 247, 2.8 min), fuhalol ([M − H]^−^ at *m*/*z* 265, 6.1 min), pentaphlorethol ([M − H]^−^ at *m*/*z* 621, 7.3 min), fucopentaphlorethol ([M − H]^−^ at *m*/*z* 745, 7.3 min) and dihydroxypentafuhalol ([M − H]^−^ at *m*/*z* 669, 8.4 min), which have all been described in other *Sargassum* species [49,50], with the exception of dibenzodioxine-1,3,6,8-tetraol, which has only been described in *F. vesiculosus* and *A. nodosum* [27,64]

Interestingly, EtOAc revealed the presence of several compounds that appear to be phlorotannin sulfate derivatives, since they all showed a common neutral loss of −80 Da, which is indicative of a SO_3_ group [65]. In more detail, the compound with [M − H]^−^ at *m*/*z* 345, eluting at 2.8, was tentatively identified as a fuhalol sulfate, since its MS/MS spectrum revealed a major product ion at *m*/*z* 265 (−80 Da), indicating a fuhalol moiety, as well as other minor product ions evidencing neutral losses that typically occur in phlorotannins (*m*/*z* 203, 219, 301 and 327 corresponding to the loss of *O*-phloroglucinol, phloroglucinol, ethylene plus water and water moiety, respectively) or are indicative of phloroglucinol units (*m*/*z* 123) and dimmers (*m*/*z* 245 and 247). Likewise, the compounds eluting at 4.5 and 4.8 min, both showing a [M − H]^−^ at *m*/*z* 469, not only revealed the same product ion at *m*/*z* 389 (−80 Da), which suggests a trifuhalol moiety, but also several other product ions at *m*/*z* 263, 245 and 139 that resulted from the combined losses of phloroglucinol plus SO_3_ (−126–80 Da), phloroglucinol plus water plus SO_3_ (−126−18−80) and phloroglucinol dimmer plus SO_3_ (−250–80), respectively. Therefore, based on their MS^2^ spectra, these two compounds were tentatively assigned as trifuhalol sulfate isomers.

Three additional compounds have been detected as possible phlorotannin sulfate derivatives at 7.0 ([M − H]^−^ at *m*/*z* 505), 9.2 ([M − H]^−^ at *m*/*z* 503) and 13 min ([M − H]^−^ at *m*/*z* 723). Although an exact identification was not achieved for these compounds, their MS/MS spectra revealed several neutral losses that are indicative of the SO_3_ group (−80 Da), phloroglucinol and phloroglucinol derivatives (e.g., −126, −142) or a combination of both, as well as the usual losses of water (−18 Da) or cross ring cleavages (−44 Da) that are common in phlorotannins. 

Although unusual, the appearance of phlorotannin sulfate derivatives in brown seaweeds is not uncommon. Indeed, such compounds have been previously described in other species, such as *Pleurophycus gardneri* [66] and *A. nodosum* [65]. Moreover, according to Emeline et al. [67], in *Littorina littorea*, upon the commencement of grazing activity, a significant increase in the gene expression of aryl sulfotransferase, an enzyme known to be involved in the sulfation of various compounds (including phenols), was observed in *F. vesiculosus*, which could be responsible for the sulfation of phlorotannins as part of its anti-grazing defense mechanism.

An additional sulfated phenolic compound was detected in this chromatogram corresponding to the most intense peak that was eluted at 1.4 min. This compound with a [M − H]^−^ at *m*/*z* 217 was identified as hydroxybenzoic acid sulfate based on its a MS^2^ spectrum, which was consistent with that previously reported for this phenolic acid [65,68]. The detection of hydroxybenzoic acid in brown seaweeds has been already reported for numerous species [69] and its sulfated derivative has been recently described in *Ascophyllum nodosum* [65].

A particularly interesting phlorotannin derivative was found at 1.8 min, showing a [M − H]^−^ at *m*/*z* 395. In addition to the typical neutral losses of 18, 126 and 142 (corresponding to water, phloroglucinol and -*O*-phloroglucinol, respectively) the MS/MS spectrum of this compound also revealed the presence of the product ion at *m*/*z* 249, which is not only indicative of a phloroglucinol dimmer but also results from the loss of 146 Da, which could be attributed to a fucose moiety. To the authors’ knowledge, there are no previous studies reporting the detection of phlorotannins linked to fucose or other sugars in brown seaweeds. However, it is well known that phlorotannins occur either in the soluble form or bound to seaweed cell walls, fulfilling important structural roles together with other cell wall components, such as fucoidan, a polysaccharide mainly composed of fucose [70]. Therefore, although this hypothesis requires further confirmation with additional spectroscopic techniques, it is possible that this compound could correspond to a phlorethol-fucoside.

Notably, even though their structural elucidation were not achieved, nine other compounds were identified as phlorotannin derivatives (Table 5) based on their MS^2^ spectra which exhibited fragmentation patterns similar to those of phlorotannins, either yielding product ions that are indicative of one or multiple phloroglucinol units and derivatives (e.g., *m*/*z* 125, 247/249, 371/373, 497, 619), or fragments that are indicative of one or multiple phloroglucinol losses (e.g., −124/126, −250, −374, −498), along with the common water losses or cross ring cleavages, and their combination with phloroglucinol units.

## 4. Conclusions

Phlorotannins have been reported to potentially provide a variety of health benefits. Indeed, these compounds have previously shown promising activities. The present study aimed to provide the chemical features of *S. vulgare* specimens collected from the Coast of the Mediterranean Sea, as well as to explore the potential bioactive properties of crude hydroacetonic extract and its purified fractions obtained from this seaweed. According to our results, both the n-hexane and ethyl acetate fractions obtained from the hydroacetonic extract of *S. vulgare* have significant potential to act as antioxidant, antidiabetic and anti-inflammatory agents. Likewise, UHPLC-DAD-ESI-MS^n^ data analysis of the latter sample allowed us to detect 21 phlorotannins, in addition to several derivatives with molecular weights ranging from 2 to 6 units of phloroglucinol, through a detailed interpretation of their fragmentation patterns. This study contributes valuable knowledge on the phlorotannin composition of *S. vulgare*, phlorotannins being potentially useful as novel functional ingredients in the development of nutraceuticals and pharmacological applications designed to treat oxidative stress, inflammation, diabetes and obesity. Hopefully, our findings will inspire future studies of *S. vulgare* that will provide a deeper understanding of the exact structural characteristics of phlorotannins, their mechanisms of action and the individual bioactive contributions of phlorotannins, as well as possible synergistic or antagonistic effects that may occur in the extracts.

## Figures and Tables

**Table 1:** Chemical composition of the freeze-dried *S. vulgare*.

Components	Amount (% DW)
Carbohydrate	68.0 ± 1.0
Ash	23.7 ± 0.1
Crude Fiber	22.9 ± 1.2
Protein content	7.70 ± 0.1
Fat content	0.51 ± 0.04

Data are expressed as means ± standard deviations.

**Table 2:** Extraction mass yield and total phlorotannin content (TPhC) of *S. vulgare* crude extract and purified fractions.

Sample	Yield (%)	TPhC (mg PGE/g ext)
Crude extract	25.9 ± 0.80 ^b^	4.7 ± 0.11 ^b^
Hex	10.8 ± 2.57 ^d^	0.4 ± 0.02 ^c^
EtOAc	6.1 ± 1.11 ^c^	9.4 ± 0.03 ^a^
AQ	63.0 ± 2.20 ^a^	0.8 ± 0.2 ^c^

Yield is expressed as *w*/*w* of dry algae (for crude extract) or of crude extract (for purified fractions). PGE—phloroglucinol equivalents; Hex—hexane fraction; EtOAc—ethyl acetate fraction; AQ—aqueous residue. Data expressed as means ± standard deviations. Different letters within a column indicate significant differences with *p* < 0.05 using Tukey’s test.

**Table 3:** Antioxidant activities of the hydroacetonic crude extract and of the purified fractions from *S. vulgare*.

Sample	DPPH^•^	ABTS^•+^	Superoxide	Galvinoxyl	β-Carotene	CUPRAC	FRAP	Phenanthroline
	IC_50_ (µg/mL)						A_0.5_ (µg/mL)	
Crude Ext	97.41 ± 2.15 ^a^	72.9 ± 5.83 ^b^	>800	364.65 ± 0.93 ^a^	65.17 ± 0.68 ^b^	303.08 ± 3.59 ^a^	>200	>200
Hex	29.77 ± 1.72 ^b^	93.76 ± 2.28 ^a^	37.07 ± 1.72 ^a^	148.7 ± 1.74 ^b^	41.29 ± 1.37 ^d^	69.04 ± 5.65 ^b^	126.5 ± 3.43 ^b^	126.14 ±1.38 ^a^
EtOAc	25.83 ± 0.54 ^C^	25.07 ± 0.21 ^c^	27 ± 0.26 ^c^	15.33 ± 0.21 ^c^	72.05 ± 0.92 ^a^	37.79 ± 0.21 ^c^	64.63 ± 3.66 ^c^	32.3 ± 0.21 ^b^
AQ	96.64 ± 2.12 ^a^	74.9 ± 4.8 ^b^	>800	>800	>800	>800	251.8 ± 5.5 ^a^	>200
BHA *	6.14 ± 0.41 ^e^	1.81 ± 0.10 ^d^	>200	5.38 ± 0.06 ^d^	0.90 ± 0.02 ^f^	6.62 ± 0.05 ^e^	7.99 ± 0.87 ^e^	2.24 ± 0.17 ^d^
BHT *	12.99 ± 0.41 ^d^	1.29 ± 0.30 ^d^	>200	3.32 ± 0.18 ^e^	1.05 ± 0.01 ^f^	8.97 ± 3.94 ^e^	>200	0.93 ± 0.07 ^d^
Tocopherol *	13.02 ± 5.17 ^d^	7.59 ± 0.53 ^d^	31.52 ± 2.22 ^b^	22.02 ± 0.03	1.79 ± 0.03 ^f^	19.92 ± 1.46 ^d^	34.93 ± 2.38 ^d^	5.78 ± 030
AA *	13.94 ± 2.81 ^d^	1.74 ± 0.10 ^d^	7.5 9 ± 1.16 ^d^	5.02 ± 0.01 ^d^	52.59 ± 1.98 ^c^	13.43 ± 0.09 ^d^	6.37 ± 0.42 ^e^	5.25 ± 0.20 ^c^

* Reference compounds; AA—ascorbic acid; BHA—butylated hydroxyanisole; BHT—butylated hydroxytoluene; Tocopherol—α-Tocopherol; Crude Ext—crude extract; Hex—hexane fraction; EtOAc—ethyl acetate fraction; AQ—aqueous residue. Values are the means of three independent assays ± SD. Means followed by different letters in the same column are significantly different at *p* < 0.05.

**Table 4:** Inhibitory ability of *S. vulgare* crude hydroacetonic extract and purified fractions towards α-amylase and pancreatic lipase activities.

	A-Amylase (IC_50_, µg/mL)	Pancreatic Lipase (IC_50_, µg/mL)
Crude Extract	>400	>1000
Hex	45.43 ± 0.22 ^b^	34.49 ± 1.37 ^a^
EtOAc	42.28 ±5.85 ^b^	17.98 ± 2.2 ^b^
AQ	>400	>1000
Acarbose	359.3 ± 8.0 ^a^	--
Orlistat *	--	0.06 ± 0.001 ^c^

* IC_50_ value for orlistat is expressed in ng/mL. Hex—hexane fraction; EtOAc—ethyl acetate fraction; AQ—aqueous residue. Values are the means of three independent assays ± SD. Means in the same column followed by different letters are significantly different at *p* < 0.05.

**Table 5:** Tentative assignment of the compounds present in the ethyl acetate fraction of *S. vulgare* extract analyzed by LC-ESI-MS/MS.

Peak	RT (min)	[M − H]^−^ (*m*/*z*)	MS/MS Ions	Tentative Assignment
1	1.4	217	181, 137, 173, 149, 179, 97	Hydroxy sulfobenzoic acid
2	1.8	191	111, 173, 147, 117, 129, 101, 87, 155	Quinic acid
3	2.2	395	179, 269, 293, 305, 377, 275, 359, 351, 253, 335, 209, 139, 249, 125, 235	Phlorethol-fucoside
		267	221, 223, 249, 231, 205, 195, 177, 169, 151, 125, 141	Phlorotannin derivative
		409	265, 291, 365 263, 319, 303, 143, 139, 347, 373, 269	Phlorotannin derivative
4	2.8	247	203, 121, 81, 229, 167	Dibenzodioxine-1,3,6,8-tetraol
		345	265, 263, 245, 205, 239, 123, 203, 219, 247, 301, 327	Fuhalol sulfate
		287	241, 249, 269, 243, 215, 197, 173, 181, 225, 251, 259, 125	Phlorotannins derivative
5	4.5	469	263, 389, 245, 265, 387, 205, 139, 425, 219, 189	Trifuhalol sulfate
6	4.8	469	263, 245, 389, 265, 387, 205, 139	Trifuhalol sulfate isomer
7	5.6	363	345, 283, 319, 237, 301, 247, 327, 273, 263, 291, 317, 221, 138, 275, 257	Phlorotannins derivative
8	6.1	265	221, 247, 139, 123, 89, 193, 237, 219, 229, 177, 191, 153, 141, 125	Fuhalol
9	7.0	505	425, 407, 289, 331, 487, 279, 215, 363, 379, 461	Phlorotannin sulfate
10	7.3	521	431, 503, 373, 255, 401, 395, 305, 477, 461, 423, 365, 247, 347, 233, 165, 229	Phlorotanin derivative
		621	481, 373, 233, 247, 497, 577, 603, 355, 437, 311, 531, 589, 283	Pentaphlorethol
		745	497, 727, 619, 353, 479, 601, 701, 371, 339, 209, 585, 229, 245	Fucopentaphlorethol
11	8.2	573	431, 359, 529, 555, 341, 323, 297, 291, 269, 217, 511, 403	Phlorotannin derivative
12	8.4	669	651, 625, 401, 465, 637, 607, 527, 579, 499, 367, 263, 299, 245	Dihydroxypentafuhalol
13	8.7	698	680, 666, 550, 432	Unkown
14	9.2	503	261, 387, 485, 423, 459, 245, 205, 297, 325, 279 359, 173, 467, 441	Phlorotannin sulfate
15	9.6	421	403, 377, 277, 345, 361, 389, 331, 303, 249, 213	Phlorotannins derivative
16	9.8	747	729, 703, 685, 621, 387, 497, 481, 607, 667, 715, 515, 359, 303, 261, 249	Phlorotannins derivative
		435	417, 403, 391, 249, 315, 207, 329, 373, 355, 345, 293, 217, 189	Phlorotannins derivative
17	10.4	407	375, 331, 313, 389, 357, 287, 345, 259	Unkown
18	10.9	577	297, 279, 192, 210, 559	Unkown
19	12.6	335	171, 317, 291, 163, 299, 275, 247, 127	Unkown
		403	327, 385, 371, 309, 341, 359, 353, 269, 293	Unkown
20	13.0	723	677, 705, 643, 583, 597, 455, 333	Phlorotannin sulfate
		755	679, 737, 692, 641, 477, 351	Unkown
		559	279, 235, 297, 192, 515, 541	Unkown
		713	677, 695, 669, 633, 449, 315	Unkown

## Data Availability

The data presented in this study are available in the article.
